# Morphological Hydrogel Microfibers with MXene Encapsulation for Electronic Skin

**DOI:** 10.34133/2021/7065907

**Published:** 2021-03-03

**Authors:** Jiahui Guo, Yunru Yu, Dagan Zhang, Han Zhang, Yuanjin Zhao

**Affiliations:** ^1^State Key Laboratory of Bioelectronics, School of Biological Science and Medical Engineering, Southeast University, Nanjing 210096, China; ^2^Department of Rheumatology Immunology, Institute of Translational Medicine, The Affiliated Drum Tower Hospital of Nanjing University Medical School, Nanjing 210008, China; ^3^Wenzhou Institute, University of Chinese Academy of Sciences, Wenzhou 325001, China

## Abstract

Electronic skins with distinctive features have attracted remarkable attention from researchers because of their promising applications in flexible electronics. Here, we present novel morphologically conductive hydrogel microfibers with MXene encapsulation by using a multi-injection coflow glass capillary microfluidic chip. The coaxial flows in microchannels together with fast gelation between alginate and calcium ions ensure the formation of hollow straight as well as helical microfibers and guarantee the *in situ* encapsulation of MXene. The resultant hollow straight and helical MXene hydrogel microfibers were with highly controllable morphologies and package features. Benefiting from the easy manipulation of the microfluidics, the structure compositions and the sizes of MXene hydrogel microfibers could be easily tailored by varying different flow rates. It was demonstrated that these morphologically conductive MXene hydrogel microfibers were with outstanding capabilities of sensitive responses to motion and photothermal stimulations, according to their corresponding resistance changes. Thus, we believe that our morphologically conductive MXene hydrogel microfibers with these excellent features will find important applications in smart flexible electronics especially electronic skins.

## 1. Introduction

Electronic skins [1–3], also known as stretchable electronic systems, are generally considered to be able to simulate the perception of human skin for various stimuli, such as deformation and temperature. Benefiting from dramatic progresses on the study of stretchable electronics, electronic skins have attracted considerable interests to both commercial development and the research community due to their potential applications in artificial intelligence systems [4, 5], wearable health monitoring [6, 7], human-machine interface [8, 9], and other fields [10–12]. Nowadays, numerous electrical conductors, including ionic liquids [13, 14], liquid metals [15, 16], and other two-dimensional materials [17–19], have been integrated with stretchable sheets to respond to external stimuli and transmit corresponding electrical signals. Among those electrical conductors, MXenes [20, 21], as one class of two-dimensional early-transition metal carbides/carbonitrides, have emerged with widespread attention due to their fascinating properties, such as large hydrophilic surfaces and excellent electrical/thermal conductivity. These MXenes have also been widely and successfully assembled into two-dimensional materials and even in three-dimensional frameworks to improve their functions [22–24]. Although with many successes, most of the MXene-derived electronic systems are with simple structures or morphologies because of their comparatively simple preparation process, which might restrict their performances under complex situations. In addition, the uncontrollable distributions of MXene in these systems usually make their surface exposed to other chemical additives, which would impair its electrical conductivity and the practical applications in flexible electronic systems. Therefore, it is still a considerable challenge to fabricate novel MXene electronic systems with elaborated structure and controllable morphology for electronic skins.

**Figure 1:**
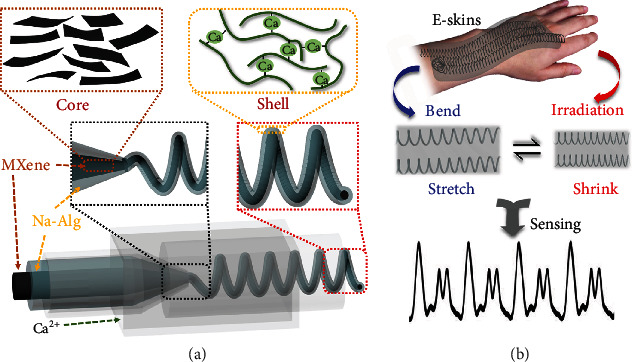
Schematic illustration of morphological hydrogel microfibers with MXene encapsulation for electronic skins. (a) Schematic diagram of the microfluidic spinning process for helical hydrogel microfibers with MXene encapsulation. (b) Scheme of the application of morphological hydrogel microfibers as electronic skins (E-skins).

In this paper, we present novel morphological hydrogel microfibers with MXene encapsulation through microfluidic spinning approach for flexible electronic skins, as schemed in Figure 1. Since microfluidics was firstly proposed by the semiconductor industry and latterly developed by the microelectromechanical system (MEMS) fields, it is defined as a special technology where fluids could be precisely manipulated by devices at micro scales [25]. Benefiting from the simple but precise control of microfluidic spinning, these technologies have been considered as a promising candidate for the fabrication of functional microfiber materials with diverse morphologies, including Janus [26], multicomponent [27], flat [28], grooved [29], hollow [30], helical [31], etc. [32–34]. Although with many successes in preparing microfibers through microfluidic technology for applications in different areas, hollow and helical hydrogel microfibers with MXene encapsulation still have not been fabricated by recent microfluidic spinning approach, and their potential values in flexible electronics especially in electronic skins remain unexplored.

Thus, we herein employed a coflow capillary microfluidic device with multiple injection channels for consecutive generation of MXene encapsulated hollow as well as helical calcium alginate (Ca-Alg) hydrogel microfibers. Both the sodium alginate (Na-Alg) and MXene solution formed laminar flows in coaxial microchannels because of their low Reynolds numbers. The rapid cross-linking reaction between Na-Alg and Ca^2+^ helped to form the core-shell structure of the microfiber and guaranteed the *in situ* encapsulation of MXene. It was found that with the increased flow ratio of inner to outer phases, microfibers could then spiral and be further solidified to maintain the helical structure. Owing to the precise control over phase flow rates and capillary diameters of microfluidic technology, hollow helical microfibers with desired morphologies can also be continuously generated. Because MXenes in an aqueous dispersion could be gelated by divalent metal ions such as Ca^2+^ [35], helical microfibers with better morphologies and more stable conductivity could be generated. Based on these morphologically conductive microfibers, we have demonstrated that they had outstanding capabilities of sensitive responses to motion and photothermal stimulations, according to their corresponding resistance changes. These performances indicated that the proposed MXene hydrogel microfibers are valuable for smart flexible electronics especially in electronic skins.

## 2. Results

In a typical experiment, a coflow microfluidic system was assembled by coaxially inserting inner spindle capillary and middle tapered injection capillary into an outer collection capillary. The microfluidic spinning method is scalable for microfluidic precursor solutions which could be gelled rapidly through UV-induced polymerization, phase inversion, and ionic or chemical cross-linking. In addition, microfluidics has been considered as a versatile fabrication tool for the generation of functional materials like nanocrystals, microparticles, microfibers, etc., with controllable sizes, shapes, and designed features. For its flexibly conductive ability, MXene (Ti_3_C_2_) was chosen as the core fluid. As MXene nanosheets have abundant surface functional groups to produce negatively charged hydrophilic surfaces, they could form a unique polymer network structure with hydrogels, significantly improving the mechanical properties of hydrogels. Meanwhile, alginate was chosen as the shell material of hydrogel microfibers to achieve a uniform shell structure for encapsulation, because of the fast gelation of sodium alginate and calcium chloride together with MXene aqueous solution and calcium ions.

**Figure 2:**
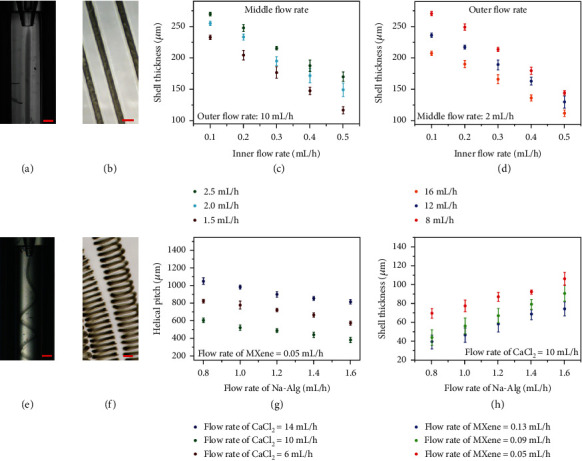
Microfluidic generation of microfibers. (a, b) Real-time microscopy image of microfluidic spinning and optical microscopic image of straight microfiber with MXene encapsulation, respectively. Scale bar: 267 *μ*m. (c, d) Relationships between shell thickness of straight microfiber and inner flow rates by tuning middle and outer flow rates, respectively. (e, f) Real-time microscopy image of microfluidic spinning and optical microscopic image of helical microfiber with MXene encapsulation, respectively. Scale bar: 307 *μ*m. (g) Relationships between helical pitches of helical microfiber and Na-Alg flow rates by tuning CaCl_2_ flow rates. (h) Relationships between shell thickness of helical microfiber and Na-Alg flow rates by tuning MXene flow rates.

When operating the microfluidic devices, the two precursors formed laminar flows at the point they pumped out from the capillaries due to the low Reynolds numbers and hydrodynamic focusing effect. After the coflow stream was introduced into the collection capillary, it was then sheathed by the outer CaCl_2_ stream that flowed from the region between the injection capillaries and the outer square capillary in a different direction. The fast diffusion of Ca^2+^ ions improved the gelation of alginate to form the shell structure of the microfibers; thus, conductive hydrogel microfibers could be continuously spun. Because MXene could be encapsulated tightly inside hydrogels, the conductivity of the produced hydrogel microfibers could as a result be stabilized (Figure 2(a)). Because the cross-linking process was very fast, the generated microfibers could replicate the coaxial construction of the microfluidic channels, and the encapsulation of MXene could take place simultaneously and successfully (Figure 2(b)). The length of the microfibers fabricated by microfluidics could achieve up to several meters. As a result, the core-shell structure of the microfibers could be easily tuned by changing different phase flow rates, as shown in Figures 2(c) and 2(d). When the outer flow rate and middle flow rate were fixed, an increase of the inner flow rate brought about a decrease of the shell thickness. Similarly, sheath thickness increased with the increase of outer flow rate as the inner and middle flow rates were fixed, while an increased middle flow rate conversely resulted in an increased shell thickness at fixed inner and outer flow rates.

Moreover, when there is a flow rate difference between the jetting stream and the surrounding fluid, the stream deforms and begins to spiral in the collection pool. Thus, by continuously increasing the sheath phase flow rate, the microfiber began deforming and gradually formed a helical structure under a random direction of rotation (Figure 2(e)). The spiraling of the microfiber could be ascribed to the large velocity difference between the microfiber stream and the outer relatively static liquid environment, and the confinement of the small space of the collection channel. Benefiting from the continuous solidification of the extruded microfibers through the CaCl_2_ stream, this helical structure can be fixed and maintained, which means that helical microfibers with core-shell structures can be spun continuously. Meanwhile, the collection pool containing CaCl_2_ aqueous solution also provides enough space for Na-Alg to cross-link with Ca^2+^ for further gelation without interfering with the formation of the helical structure. Thanks to the simultaneous process of solidification, spiraling, and encapsulation, the solid microfibers could maintain the helical and core-shell structure. Under the optical observation, it could be seen that the collected helical microfibers had a free-standing helical geometry and a core-shell structure at the same time (Figure 2(f)). By tuning rates of MXene and Na-Alg as well as CaCl_2_ flows, the helical pitches and shell thickness of the solidified helical microfibers can be precisely regulated (Figures 2(g), 2(h), and S1). It can be concluded that the increased Na-Alg phase flow rates and decreased CaCl_2_ phase flow rates contributed to the decreased helical pitches of the microfibers. In addition, at a fixed CaCl_2_ flow rate, the increase of the Na-Alg flow rate and the decrease of the MXene flow rate brought about the increase of shell thickness. Thus, the core-shell structured helical microfibers with desired morphologies could be continuously spun under proper flow rates conditions.

**Figure 3:**
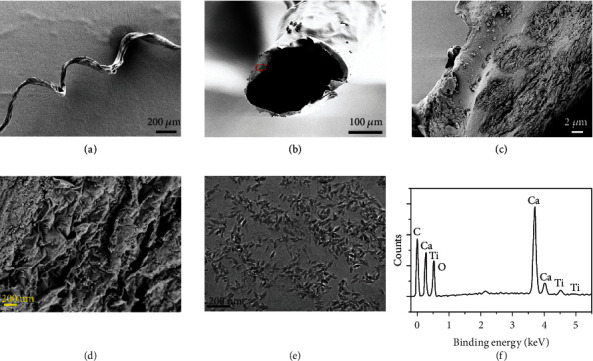
Characterization of morphological hydrogel microfibers. (a–d) SEM images of the (a) helical structured microfiber, (b) the core-shell structure of the microfiber, and (c, d) MXenes inside the fabricated microfiber at different magnification degrees. (e) TEM image of MXene aqueous solution used in microfluidic spinning process. (f) EDS results of the MXene encapsulated microfiber.

For testing the construction of derived hydrogel microfibers at nanoscale, these microfibers were investigated by scanning electron microscopy (SEM), as shown in Figures 3(a) and 3(b). It was found that the microfiber had a uniform helical geography and a core-shell cross-sectional structure due to the continuous spinning and filling process, and thus, it was believed that MXene could be successfully encapsulated in the microfiber during fabrication. This could be proven from the observation of MXene located in the core of the microfiber, as shown in Figures 3(c) and 3(d). It could be found that internal MXene flaked in microfiber inner surface, indicating that they were perfectly encapsulated and maintained their initial layered structure, which was also characterized under transmission electron microscope (TEM) in their solution state (Figure 3(e)). It could be seen that MXene exhibited a uniform thin film with wheat particulate grain structure of approximately an average size of 25 nm. Moreover, the successful encapsulation could also be confirmed through the *in situ* element analysis by Energy Dispersive Spectrometer (EDS) and Raman spectra characterization, as shown in Figures 3(f) and S2. As shown in Figure 3(f), the element of Ti, which was a unique element of MXene, was successfully confirmed to exist in the hydrogel microfibers. In Raman spectra, the peaks at 208 and 720 cm^−1^ were the symmetrical out-of-plane vibrations of Ti and C atoms, respectively. Also, Fourier Transform Infrared Spectroscopy (FTIR) measurement was also conducted to characterize the functional group of the as-prepared microfibers (Figure S3). The absorption spectra of microfibers exhibit a strong and broad band at approximately 3450 cm^−1^, which can be attributed to the stretching vibration of hydroxyl (O-H). The absorption peaks at 1093, 1384, and 1637 cm^−1^ are ascribed to the presence of C-H, COO-, and C=O of sodium carboxyl groups, respectively, indicating the successful preparation of the MXene encapsulated microfibers.

As MXene is reported with excellent electron transmission ability, the morphological hydrogel microfiber could be endowed with conductivity, and their conductive performance was tested firstly. According to the law of resistance R = *ρL*/*S*, where *ρ*, *L*, and *S* refer to the resistivity of MXene, length of microfiber, and the cross-sectional area of the inner core, respectively, microfibers with different morphologies would show various conductive performances. After measuring the as-generated straight microfibers with different core sizes and lengths (Figure S4(a) and S4(b)), it could be found that the shorter microfibers with larger core diameter showed a smaller resistance, indicating better conductivities. In particular, the microfiber at a core diameter of 185 *μ*m and a length of 2 cm showed the actual resistance at 77 k*Ω*, indicating the conductivity value at 9.7 S/m. Similarly, this situation occurred on helical microfibers with different helical pitches and lengths, as shown in Figure S4(c) and S4(d). When the length of helical microfibers was fixed, a larger helical pitch would bring about a smaller resistance, because the actual length of microfibers was shorter than those of microfibers with larger helical pitches. Conversely, when helical pitches of microfibers were determined, an increase of length resulted in an increase of resistance, which was consistent with the law of resistance.

**Figure 4:**
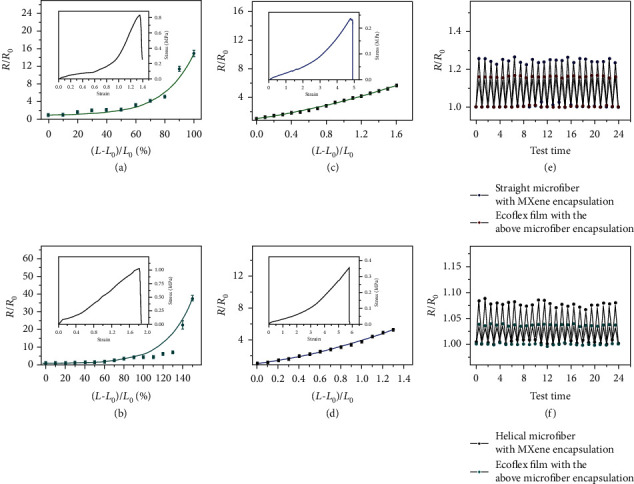
Conductivity performances of microfibers under stretch. (a) Relative resistance changes when stretching the straight microfiber. Inset: stress-strain curve of straight microfiber. (b) Relative resistance changes when stretching the helical microfiber. Inset: stress-strain curve of helical microfiber. (c) Relative resistance changes when stretching the films with straight microfiber encapsulation. Inset: stress-strain curve of the film with straight microfiber encapsulation. (d) Relative resistance changes when stretching the films with helical microfiber encapsulation. Inset: stress-strain curve of the film with helical microfiber encapsulation. (e) Cycled tests of the relative resistance change of straight microfibers and films with straight microfiber encapsulation under 20% stretch. (f) Cycled tests of the relative resistance change of helical microfibers and films with helical microfiber encapsulation under 20% stretch.

Based on these characterizations of basic electrical properties, the conductive performances of the fabricated microfibers under stretching process should be anticipated. Before checking the conductivities, the mechanical property of hollow microfibers with MXene encapsulation was first tested to see whether these microfibers could be stretched. As shown in Figure 4(a), inset, the microfibers could achieve a stretchability of approximately 130% owing to the hydrogel shell, while pure hydrogel microfibers obtained about 138% stretchability (Figure S5). The responsibility during the stretching process was then assessed by analyzing its relative resistance *R*/*R*_0_, where *R*_0_ was the initial resistance and *R* was the resistance under varying strains. The increasing relative resistance from the curve suggested that the increase of the length and the decrease of the cross-sectional area brought about a larger resistance during stretching. However, the increase of the resistance was not as much in the helical microfiber stretching process (Figure 4(b)), and the stretchability of helical microfibers could be up to about 180% (Figure 4(b), inset). This was because at the very beginning of the stretching process, the length change was negligible, and thus, the resistance changes mainly depended on the variation of the cross-sectional area of the MXene core. Until the helical microfiber began to be stretched to be straight, the increased strain leads to an elongation of microfiber as well as a shrinkage of its cross-sectional area, causing a remarkable increase in the relative resistance change.

To evaluate the practical value of the generated conductive microfibers as strain sensors, they were further embedded in a flexible polymer film as a paradigm to prototype stretchable electronics. Compared with pure microfibers, the composite film could achieve a great stretchability of approximately 500% (Figure 4(c), inset). The conductive performance during stretching was similarly carried out, as shown in Figure 4(c). In contrast with the relative resistance variation *R*/*R*_0_ of pure microfibers, that of composite film increased slightly, which might be owing to the fact that the changes in the cross-sectional area of microfibers were limited in the film during the stretching process. The similar situation also occurred during the electrical test for composite films embedded with helical microfibers (Figure 4(d)). The variation of its resistance only achieved 5.2 times of *R*_0_ when stretched to 130%, while the stiffness of the composite film with helical microfibers could achieve a stretchability of 570% (Figure 4(d), inset). The presented resistance change was around 140-160%, because fibers would be dehydrated in the process of film curing. During stretching, fibers encapsulated in films would fracture and the degree of tensileness is usually between ~150 and 200%. Additionally, the hollow as well as helical microfibers and the composite films were cycle tested to confirm their stability. As depicted in Figures 4(e) and 4(f), under the condition of 20% stretch for 24 times, there was no significant break in the resistance change of various test elements, which proved that those microfibers and composite films owned an outstandingly stable and repeatable conductivity to strains in practical applications.

**Figure 5:**
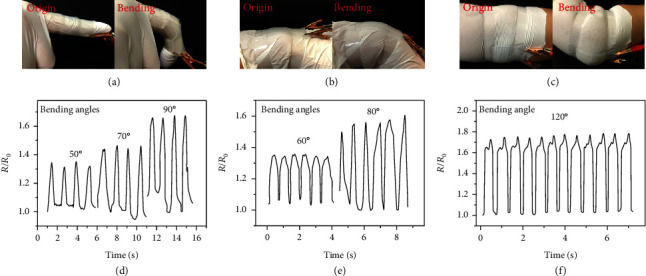
Conductivity response to various human motions in real time. (a–c) Digital images of the flexible film responding to bending motions of the finger, wrist, and elbow, respectively. (d–f) Relative resistance changes of the flexible film responding to bending motions of the finger, wrist, and elbow at different bending angles, respectively.

To take it one step further, those composite films embedded with morphologically conductive microfibers were taken as electronic skins for real-time detection of human activities. For example, human joint motion could be easily identified by recording the relative resistance changes of the sensor in a highly repeatable manner (Figure 5(a)). Firstly, the integrated flexible film of microfibers was attached on the finger and its real-time electrical signals were recorded. During the to-and-fro bending of fingers, the relative resistance changes showed an obvious and regular increase and decrease, which also varied according to the degree of the finger bending, as shown in Figure 5(d). Apart from fingers, the motions of the wrist and elbow are also detected (Figures 5(b) and 5(c)). By sticking the integrated film on a wrist or elbow support, the motions could be studied by corresponding resistance changes (Figures 5(e) and 5(f)). It could be inferred that the resistance change of the integrated film would be significantly different as the range of motion varied. This proved that the flexible and durable composite film based on morphologically conductive hydrogel microfibers with MXene encapsulation was promising for real-time monitoring of human motions.

**Figure 6:**
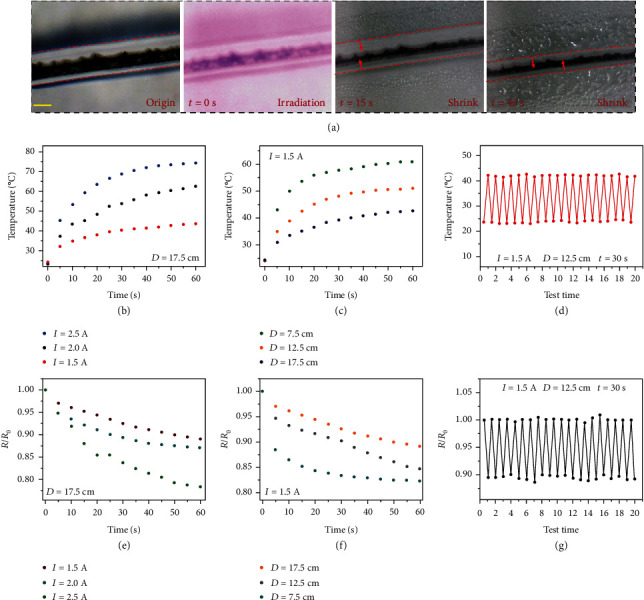
Temperature and conductivity responses to NIR irradiation in real time. (a) Photographs of the shrinkage of the straight microfiber with the increasing temperature. Scale bar: 210 *μ*m. (b, c) Relationships between the temperature and the irradiation time at different irradiation powers and irradiation distances, respectively. (d) Cycled tests of the relative temperature changes after irradiation. (e, f) Relationships between the resistance changes and the irradiation times at different irradiation powers and irradiation distances, respectively. (g) Cycled tests of the relative resistance changes after irradiation.

Furthermore, the outstanding photothermal characteristic of MXene imparted the resultant morphological hydrogel microfibers with photothermal and even photoelectrical conversion abilities, two of which could be potentially used in wearable electronic skins for sensing light. After wrapping straight hydrogel microfibers within PNIPAM which was one thermal responsiveness hydrogel, those hydrogel microfibers were exposed under continuous irradiation with an 808 nm near-infrared (NIR) laser in an aqueous environment to study the variations in temperature and resistance. As shown in Figure S5, microfibers with MXene encapsulation exhibited an obvious photothermal conversion behavior, reaching a nearly identical saturated temperature of approximately 42.8°C within 60 seconds. In contrast, the temperature of pure Ca-Alg microfibers showed no significant change under the same conditions (Figure S6), indicating that the photothermal performance of MXene nanosheets was well retained in composite fibers. Meanwhile, due to the volume transition ability of PNIPAM hydrogel responding to temperature variations, the shrinking process of the composite hydrogel microfibers was observed under the light microscope (Figure 6(a)). It was shown that the shrinkage of PNIPAM caused the entire microfiber diameter to narrow, resulting in tighter MXene core contact.

Attractively, during the process, the hybrid hydrogel film could not only present temperature changes but also feed electrical resistance changes back. To be specific, in view of the photothermal conversion performances of hydrogel microfibers with different MXene content encapsulation, relationships between irradiation time and temperature changes of single composite microfibers under different irradiation powers as well as distances were studied (Figures 6(b) and 6(c)). It was found that with the enhancement of NIR laser power densities, the equilibrium temperature of hydrogel microfibers exhibited an obvious rising trend. The maximum temperature reached approximately 75°C at a laser power current of 2.5 A after approximately 60 s irradiation at the distance of 17.5 cm. Because the photothermal response of the composite microfibers became more obvious with the decrease of distance, photothermal performances of microfibers with MXene encapsulation could be readily controlled by regulating the NIR laser power densities as well as the distance between the laser probe and microfibers. Moreover, the cycle property of the photothermal effect of microfibers was also studied, as demonstrated in Figure 6(d). After 20 times of heating and cooling behaviors, the photothermal performance of microfibers showed no obvious change, proving its recyclability, laying the foundations for practical applications in wearable electronic skins.

In accordance to temperature changes, resistance variations of microfibers directly caused by PNIPAM shrinkage were studied under different irradiation powers as well as distances (Figures 6(e) and 6(f)). It was found that the relative resistance showed a negative correlation with the increase of irradiation powers or the decrease of irradiation distances under the same irradiation duration, which corresponded with the temperature changes. This should be attributed to the volume transition displayed by PNIPAM under increased temperature brought about the closer distance of MXene cores, which facilitated the transfer of electrons to eventually reduce the electrical resistance. In addition, it should be noted that the thermal response performance reflected in the resistance changes of microfibers was similarly reversible after 20 times of heating and cooling behaviors (Figure 6(g)). It suggested that the resistance responded stably and reversibly, revealing excellent reproducibility and durability of hydrogel microfibers with MXene encapsulation. All these results demonstrated that the morphologically conductive hydrogel microfibers exhibited awesome photothermal and photoelectrical conversion properties, which were of great importance and potential in the field of flexible electronic skin in the near future.

## 3. Discussion

In summary, we have generated novel morphologically conductive hydrogel microfibers with MXene encapsulation through a coaxial-flow microfluidic spinning approach. The axial symmetry aligning of microchannels as well as rapid gelation between alginate and calcium ions allowed the formation of hollow straight together with helical microfibers and guaranteed the *in situ* encapsulation of MXene. Benefiting from the easy manipulation of phases in microfluidic channels, the resultant hollow straight and helical microfibers were with highly controllable morphologies and package features. These morphologically conductive microfibers have then been investigated to reveal sensitive response to motion and photothermal stimulations, according to their relevant resistance changes. Thus, these excellent features persuade us that the fabricated novel morphologically conductive hydrogel microfibers with MXene encapsulation will find more important applications in wearable and portable electronics, especially in electronic skins.

## 4. Materials and Methods

### 4.1. Materials

The MXene aqueous suspension (Ti_3_C_2_, 5 mg/mL) was purchased from Xiyan New Material Technology Co., Ltd. (Shandong, China). Sodium alginate (low viscosity) was bought from Alfa Aesar. Calcium chloride (anhydrous), N-isopropylacrylamide (NIPAM), 2-hydroxy-2-methyl-1-phenyl-1-241 propanone (HMPP), and N,N-methylene-bisacrylamide (BIS) were purchased from Sigma-Aldrich. Water with a resistivity of 18.2 M*Ω*·cm was acquired from a Millipore Milli-Q system. Ecoflex® 00-30 was purchased from Smooth-On, Inc. (Macungie, PA). All of the chemicals were used as received unless otherwise indicated.

### 4.2. Microfluidics

Glass capillaries with different shapes were coaxially assembled on glass slides to fabricate the capillary microfluidic devices. Round glass capillary tubes with outer and inner diameters of 1.0 mm and 800 *μ*m (World Precision Instruments) were tapered to the orifice of approximately 100 *μ*m or 250 *μ*m using a capillary puller (Sutter Instrument, P-97); the inner diameter of the outlet of the glass capillary tubes with spindle tips were about 80 *μ*m or 150 *μ*m. The spindle capillary was assembled coaxially into the tapered one as the inner flow channel, and both of the two capillaries were coaxially aligned in the collection capillary along the same direction and acted as the core and shell injection channels in the microfluidics. These tapered and collection capillaries were then coaxially inserted into a square capillary with its inner diameter 1.05 mm (AIT Glass) for observation. Finally, a transparent epoxy resin was used to seal the capillaries where necessary.

### 4.3. Preparation of Microfibers with MXene Encapsulation

The spinning process was carried out in the coflow microfluidic device. The inner phase of the MXene aqueous suspension was pumped through the spindle capillary; the middle phase of 3 wt % Na-Alg and the outer phase of 2 wt % CaCl_2_ stream were pumped, respectively, in the tapered injection and collection capillary. The generated microfibers were polymerized in the collection channel and collected in glass vials containing calcium chloride solution. All fluids were pumped into the capillary microfluidic device using syringe pumps (Longer Pump LSP01). The inner flow rates could be changed in 0.05-0.5 mL/h, the middle flow rates could be 0.8-2.5 mL/h, and the outer flow rate could be varied from 6 to 16 mL/h. To conduct the photothermal effect test, a PNIPAM pregel was firstly prepared. Briefly, monomer NIPAM and cross-linked BIS were mixed at the weight ratio of 29 : 1 and the final concentration of the solution was 10 wt %. Then, photoinitiator HMPP (1%, *v*/*v*) were added in the above mixture solution. After microfibers were coated by the mixed pregel solution, UV light was used to solidify the solution to form shrinkable microfibers.

### 4.4. Preparation of Flexible Films Integrating Microfibers with MXene Encapsulation

Two components of Ecoflex® 00-30 were mixed in equal volume and homogenized, and then, the mixture was uniformly coated on morphological microfibers with MXene encapsulation, which were firstly cut into a suitable length to prevent the leaking of inner MXene. The composite was gelatinized at room temperature for about 1 hour with negligible shrinkage.

### 4.5. Electrical Tests

The characterizations on resistance and real-time resistance change of the microfibers as well as elastic films were carried out by using a digital multimeter (DMM6500, Keithley, Beaverton, USA). The electrical conductivities of hydrogels were measured by using a traditional two-probe technique. Both ends of the microfiber were connected to the probe of the electrical test system, and the specified procedure was then selected on the system.

### 4.6. Characterization

The microfluidic generation processes in the capillary microfluidic devices were snapped by a fast camera (F032B, Pike, Germany). Bright-field microscopic images were taken by a microscope (JSZ6S, Jiangnan novel optics) equipped with CCD cameras (Oplenic Digital Camera). The microstructures of microfibers were characterized by a field emission scanning electron microscope (FESEM, Ultra Plus, Zeiss). Transmission electron microscope (TEM) images were obtained through a transmission electron microscope (JEOL, JEM-2100). The infrared spectra were collected with a Thermo Scientific Nicolet iS50 Fourier Transform Infrared Spectrometer (FTIR), and Raman spectroscopy was conducted under a Raman microscope (Raman, InVia, Renishaw) with a 785 nm laser. The stiffness of microfibers was characterized by a tensile testing machine (HSV-500, HANDPI). The photothermal effect of microfibers with MXene encapsulation was tested with near-infrared irradiation (NIR, 808 nm, Xilong Tech Co., Ltd., China) and recorded by the uncooled handheld IR camera (FLIR Systems AB).

## Supplementary Materials

Supplementary Materials Figure S1: optical microscopic images of helical microfibers with different helical pitches. Scale bar: 450 *μ*m. Figure S2: the Raman spectra of Ca-Alg microfibers and Ca-Alg microfibers coated with MXene. Figure S3: the FTIR spectra of Ca-Alg microfibers and Ca-Alg microfibers with MXene encapsulation. Figure S4: conductivity performances of microfibers. Figure S5: stress-strain curve of the pure straight microfiber. Figure S6: the progress of straight microfibers with MXene encapsulation under NIR irradiation. Figure S7: the progress of straight microfibers without MXene encapsulation under NIR irradiation.
