# Supplementary material for: Morphological Hydrogel Microfibers with MXene Encapsulation for Electronic Skin
Source: Research (Wash D C). 2021 Mar 3;2021:7065907. doi: 10.34133/2021/7065907 (PMC7953990; doi:10.34133/2021/7065907)
Supplement: Supplementary Materials — Figure S1: optical microscopic images of helical microfibers with different helical pitches. Scale bar: 450 μm. Figure S2: the Raman spectra of Ca-Alg microfibers and Ca-Alg microfibers coated with MXene. Figure S3: the FTIR spectra of Ca-Alg microfibers and Ca-Alg microfibers with MXene encapsulation. Figure S4: conductivity performances of microfibers. Figure S5: stress-strain curve of the pure straight microfiber. Figure S6: the progress of straight microfibers with MXene encapsulation under NIR irradiation. Figure S7: the progress of straight microfibers without MXene encapsulation under NIR irradiation. [file 7065907.f1.zip › Supplementary Materials.docx]

Supplementary Materials


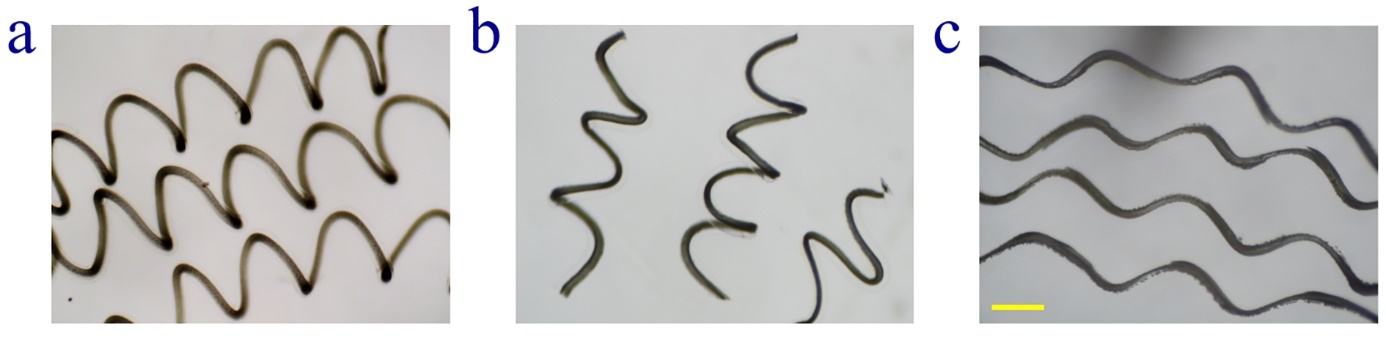


**Figure S1. Optical microscopic images of helical microfibers with different helical pitches. Scale bar: 450 μm.**


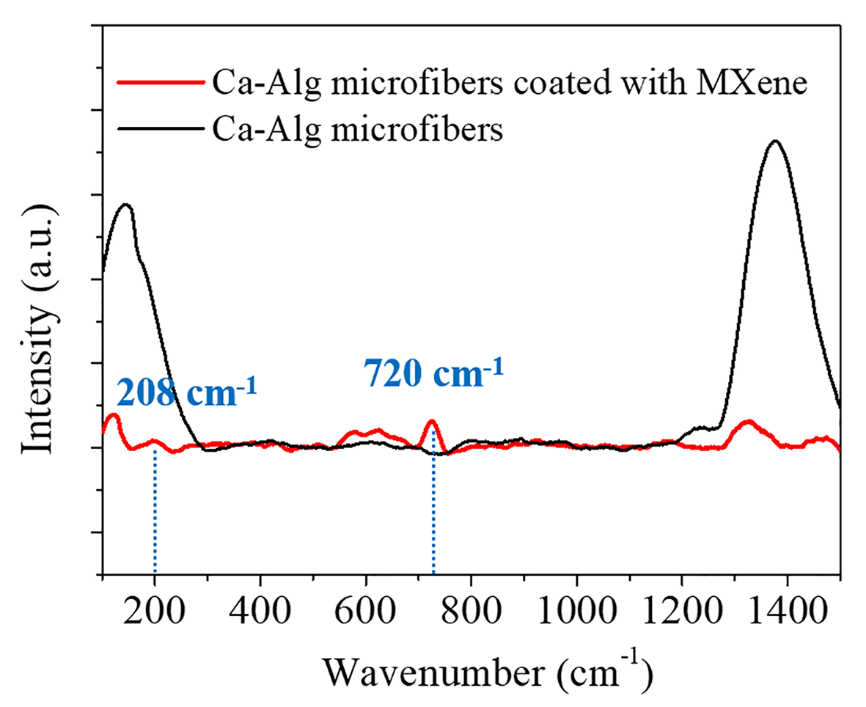


**Figure S2. The Raman spectra of Ca-Alg microfibers and Ca-Alg microfibers coated with MXene.**


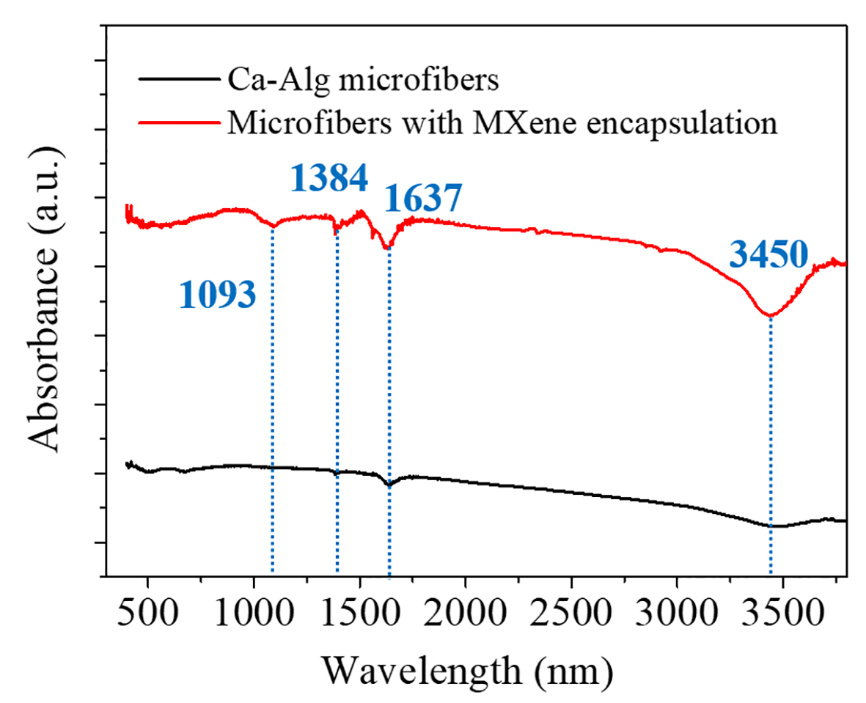


**Figure S3. The FTIR spectra of Ca-Alg microfibers and Ca-Alg microfibers with MXene encapsulation.**


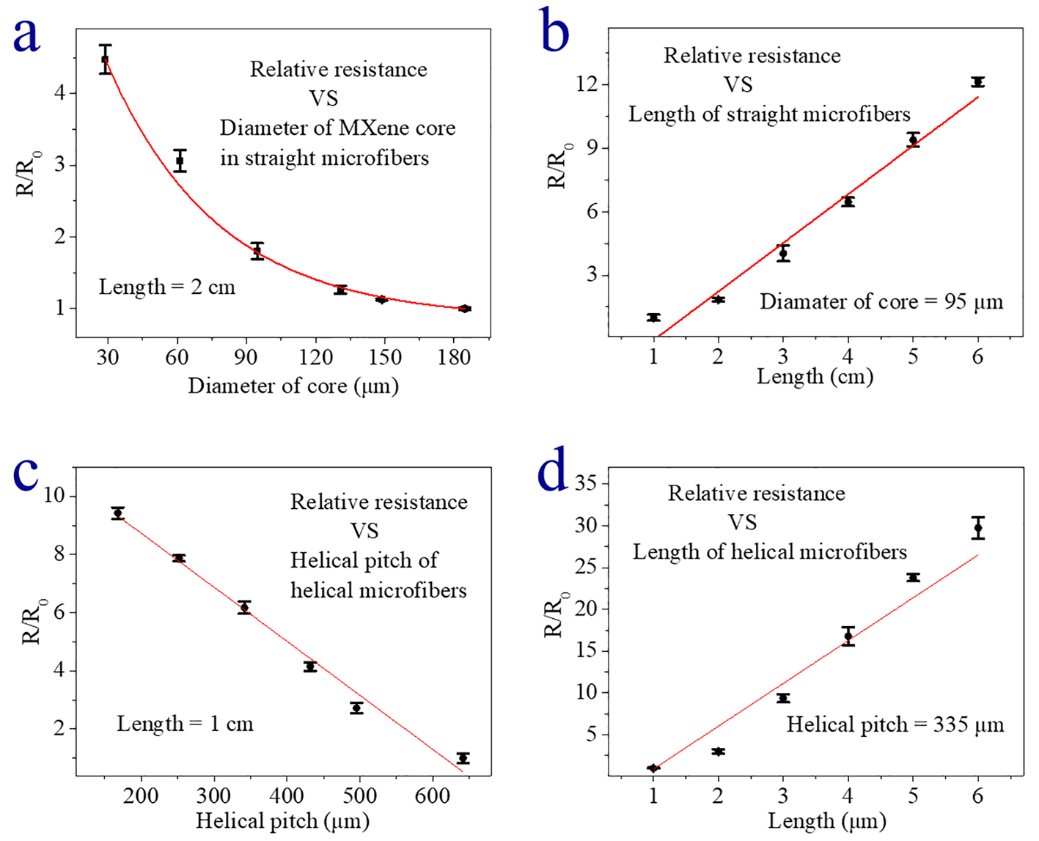


**Figure S4. Conductivity performances of microfibers.** a) Relationship between relative resistance changes and the diameter of straight microfiber’s core. b) Relationship between relative resistance changes and the length of straight microfiber. c) Relationship between relative resistance changes and the helical pitches of helical microfibers. d) Relationship between relative resistance changes and the length of helical microfibers.


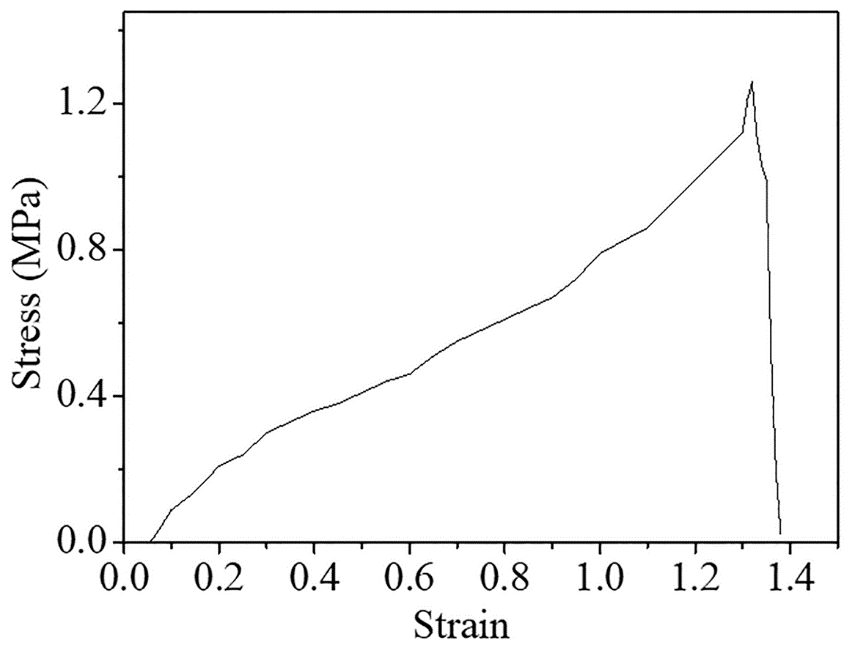


**Figure S5. Stress-strain curve of the pure straight microfiber.**


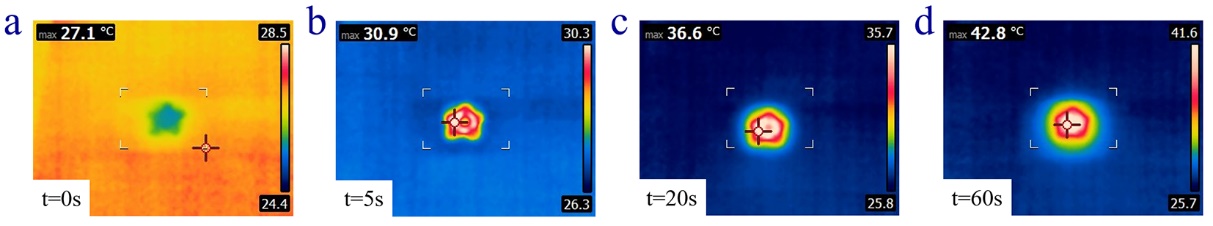


**Figure S6. The progress of straight microfibers with MXene encapsulation under NIR irradiation.**


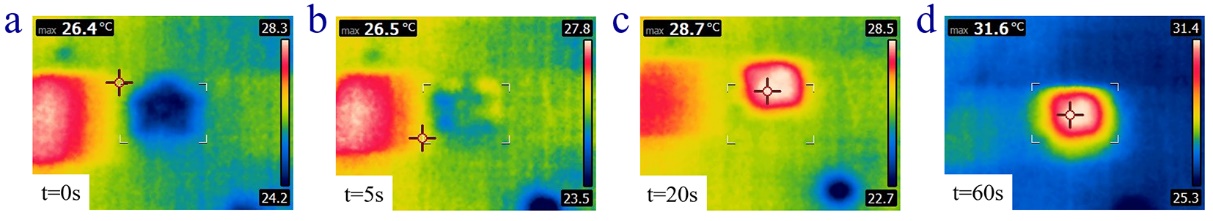


**Figure S7. The progress of straight microfibers without MXene encapsulation under NIR irradiation.**
