# Supplementary figures and images for: Morphological Hydrogel Microfibers with MXene Encapsulation for Electronic Skin
Source: Research (Wash D C). 2021 Mar 3;2021:7065907. doi: 10.34133/2021/7065907 (PMC7953990; doi:10.34133/2021/7065907)

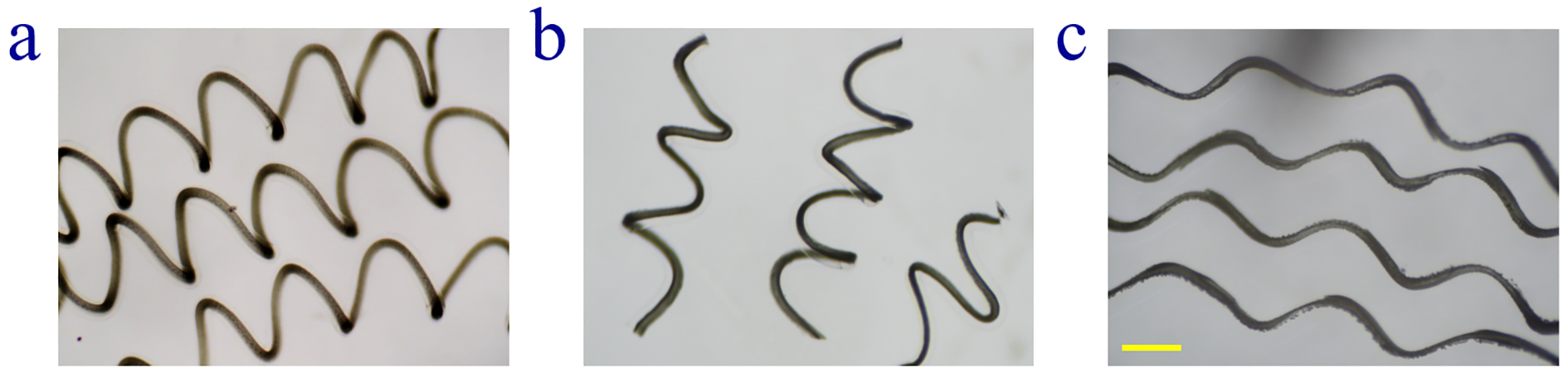

Supplement: Supplementary Materials — Figure S1: optical microscopic images of helical microfibers with different helical pitches. Scale bar: 450 μm. Figure S2: the Raman spectra of Ca-Alg microfibers and Ca-Alg microfibers coated with MXene. Figure S3: the FTIR spectra of Ca-Alg microfibers and Ca-Alg microfibers with MXene encapsulation. Figure S4: conductivity performances of microfibers. Figure S5: stress-strain curve of the pure straight microfiber. Figure S6: the progress of straight microfibers with MXene encapsulation under NIR irradiation. Figure S7: the progress of straight microfibers without MXene encapsulation under NIR irradiation. [file 7065907.f1.zip › Figure S1.png]

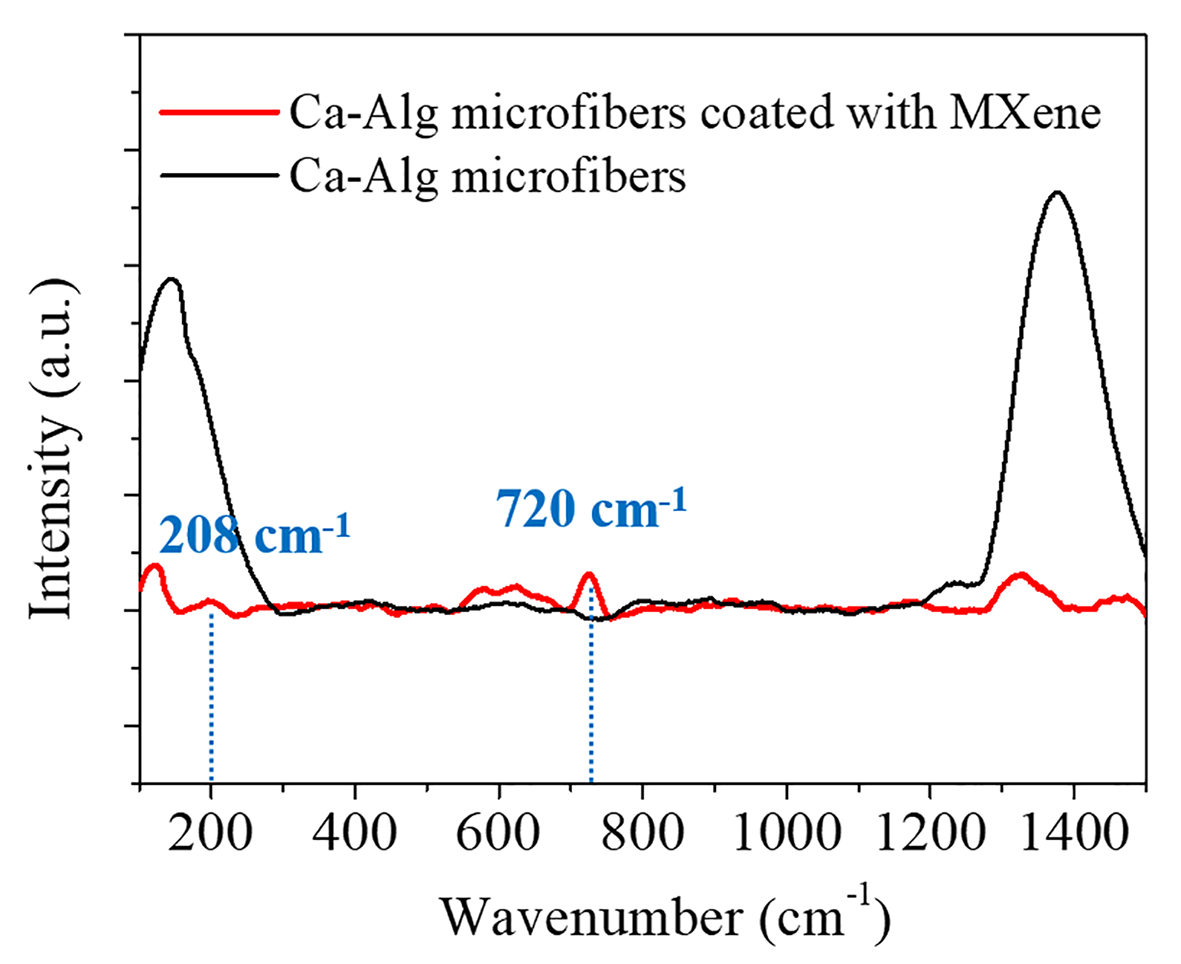

Supplement: Supplementary Materials — Figure S1: optical microscopic images of helical microfibers with different helical pitches. Scale bar: 450 μm. Figure S2: the Raman spectra of Ca-Alg microfibers and Ca-Alg microfibers coated with MXene. Figure S3: the FTIR spectra of Ca-Alg microfibers and Ca-Alg microfibers with MXene encapsulation. Figure S4: conductivity performances of microfibers. Figure S5: stress-strain curve of the pure straight microfiber. Figure S6: the progress of straight microfibers with MXene encapsulation under NIR irradiation. Figure S7: the progress of straight microfibers without MXene encapsulation under NIR irradiation. [file 7065907.f1.zip › Figure S2.png]

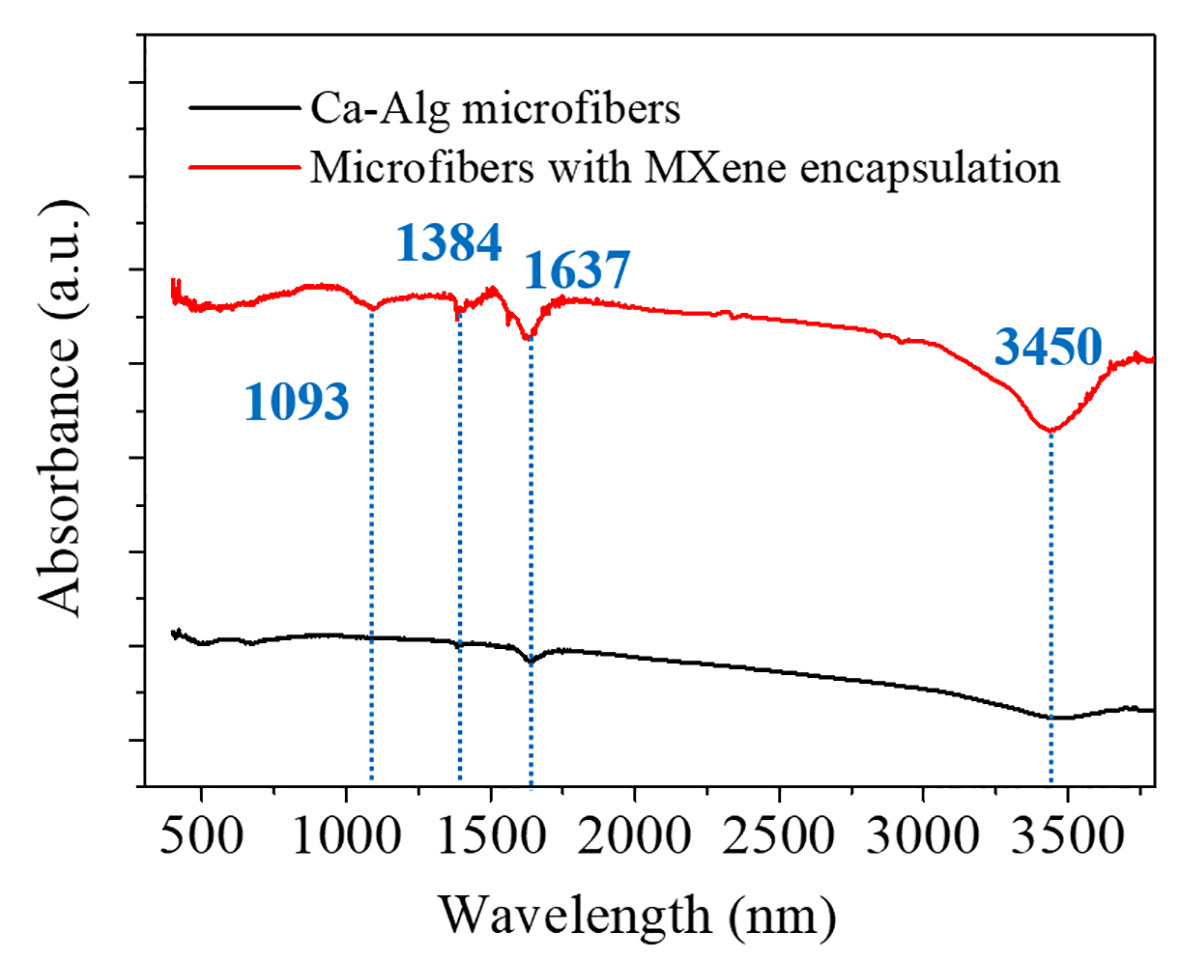

Supplement: Supplementary Materials — Figure S1: optical microscopic images of helical microfibers with different helical pitches. Scale bar: 450 μm. Figure S2: the Raman spectra of Ca-Alg microfibers and Ca-Alg microfibers coated with MXene. Figure S3: the FTIR spectra of Ca-Alg microfibers and Ca-Alg microfibers with MXene encapsulation. Figure S4: conductivity performances of microfibers. Figure S5: stress-strain curve of the pure straight microfiber. Figure S6: the progress of straight microfibers with MXene encapsulation under NIR irradiation. Figure S7: the progress of straight microfibers without MXene encapsulation under NIR irradiation. [file 7065907.f1.zip › Figure S3.png]

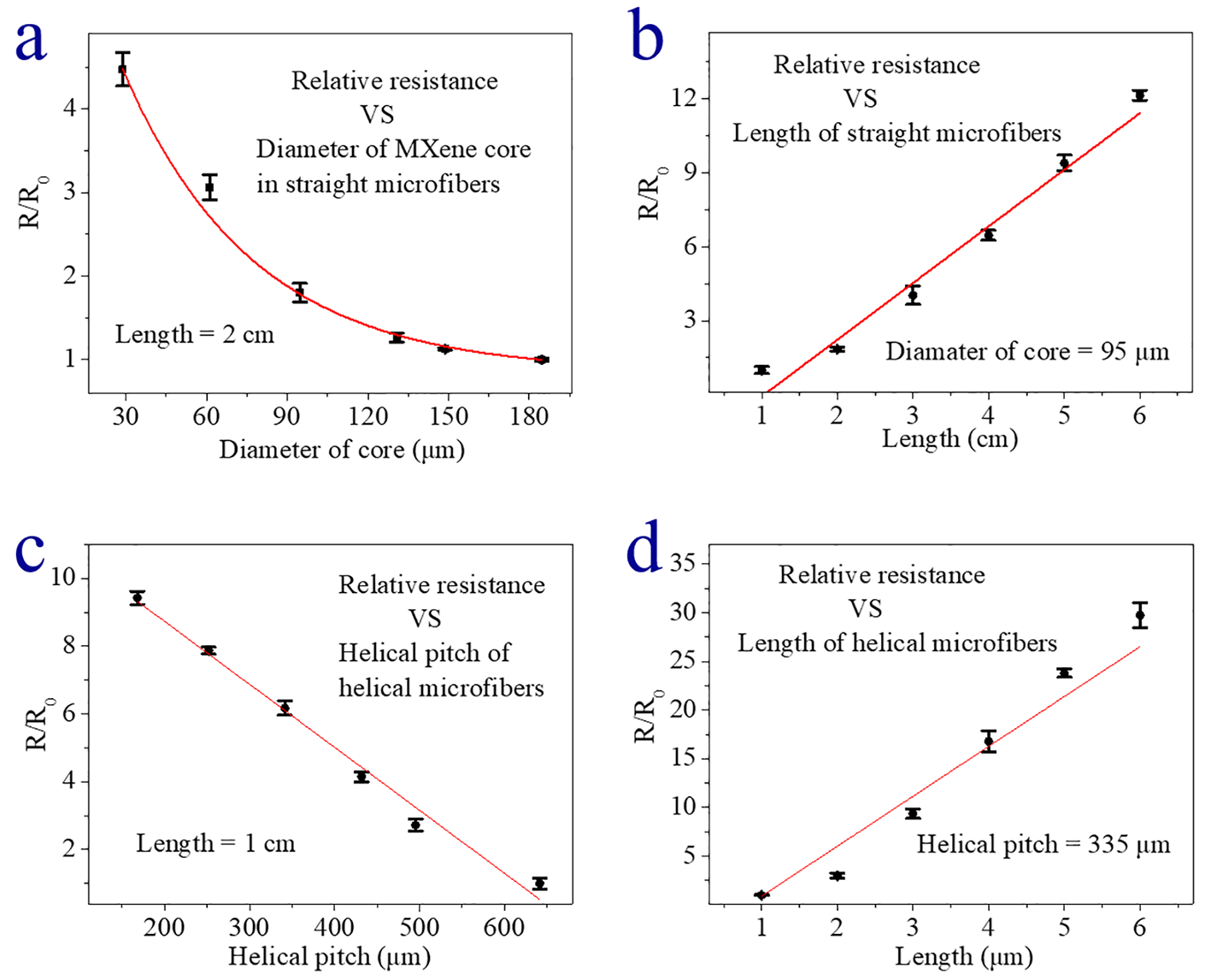

Supplement: Supplementary Materials — Figure S1: optical microscopic images of helical microfibers with different helical pitches. Scale bar: 450 μm. Figure S2: the Raman spectra of Ca-Alg microfibers and Ca-Alg microfibers coated with MXene. Figure S3: the FTIR spectra of Ca-Alg microfibers and Ca-Alg microfibers with MXene encapsulation. Figure S4: conductivity performances of microfibers. Figure S5: stress-strain curve of the pure straight microfiber. Figure S6: the progress of straight microfibers with MXene encapsulation under NIR irradiation. Figure S7: the progress of straight microfibers without MXene encapsulation under NIR irradiation. [file 7065907.f1.zip › Figure S4.png]

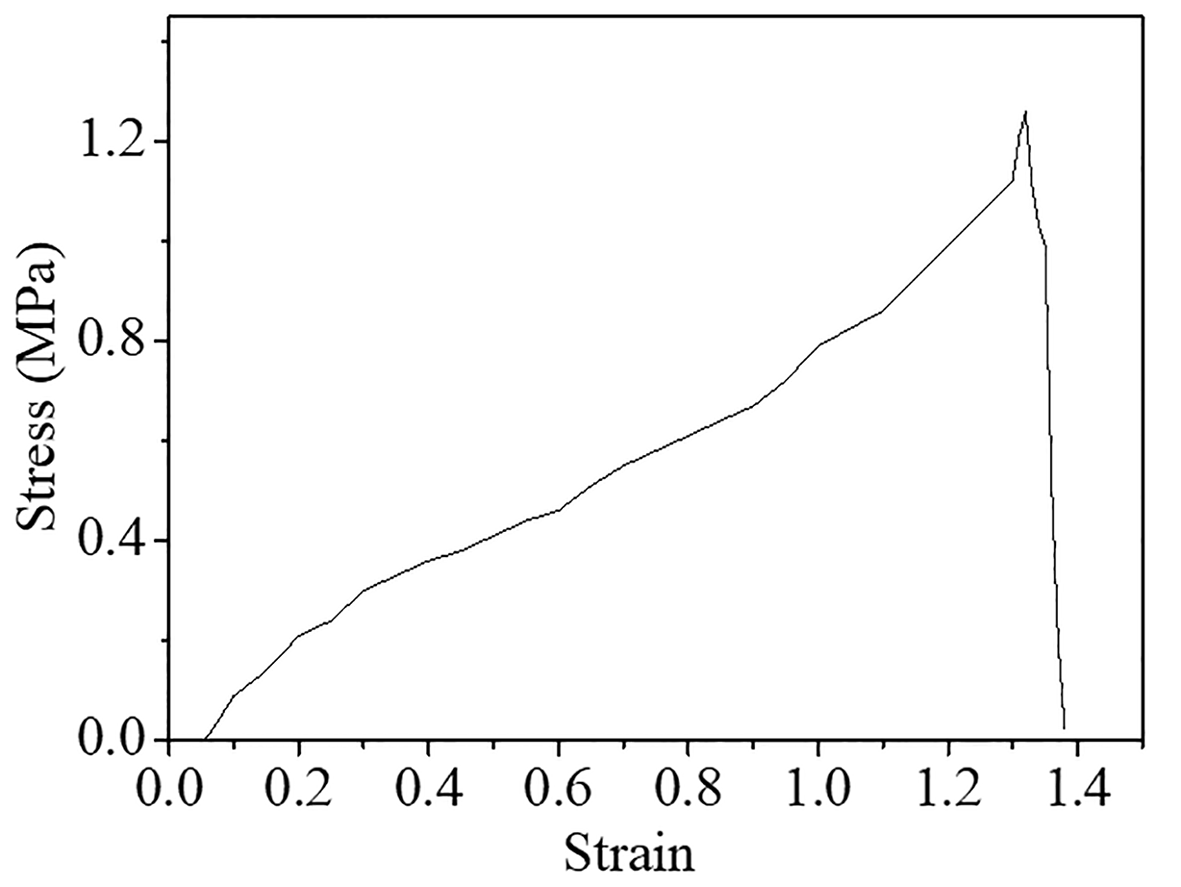

Supplement: Supplementary Materials — Figure S1: optical microscopic images of helical microfibers with different helical pitches. Scale bar: 450 μm. Figure S2: the Raman spectra of Ca-Alg microfibers and Ca-Alg microfibers coated with MXene. Figure S3: the FTIR spectra of Ca-Alg microfibers and Ca-Alg microfibers with MXene encapsulation. Figure S4: conductivity performances of microfibers. Figure S5: stress-strain curve of the pure straight microfiber. Figure S6: the progress of straight microfibers with MXene encapsulation under NIR irradiation. Figure S7: the progress of straight microfibers without MXene encapsulation under NIR irradiation. [file 7065907.f1.zip › Figure S5.png]

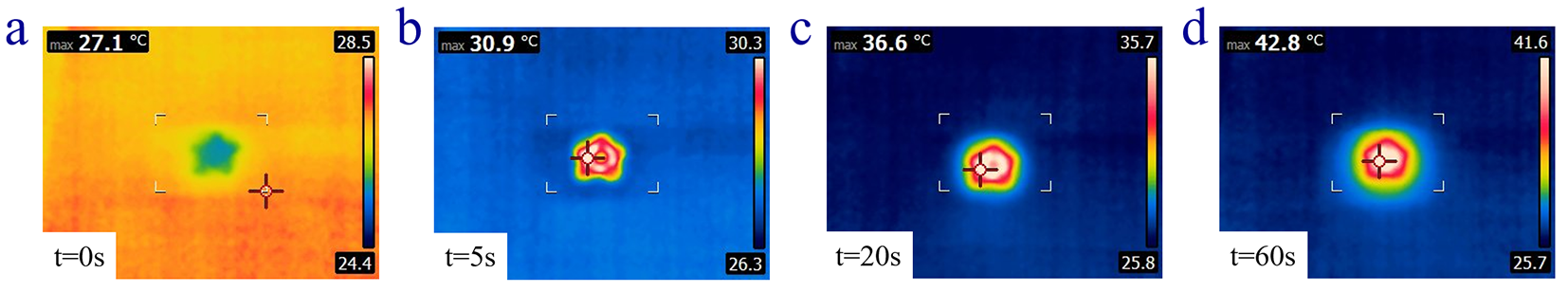

Supplement: Supplementary Materials — Figure S1: optical microscopic images of helical microfibers with different helical pitches. Scale bar: 450 μm. Figure S2: the Raman spectra of Ca-Alg microfibers and Ca-Alg microfibers coated with MXene. Figure S3: the FTIR spectra of Ca-Alg microfibers and Ca-Alg microfibers with MXene encapsulation. Figure S4: conductivity performances of microfibers. Figure S5: stress-strain curve of the pure straight microfiber. Figure S6: the progress of straight microfibers with MXene encapsulation under NIR irradiation. Figure S7: the progress of straight microfibers without MXene encapsulation under NIR irradiation. [file 7065907.f1.zip › Figure S6.png]

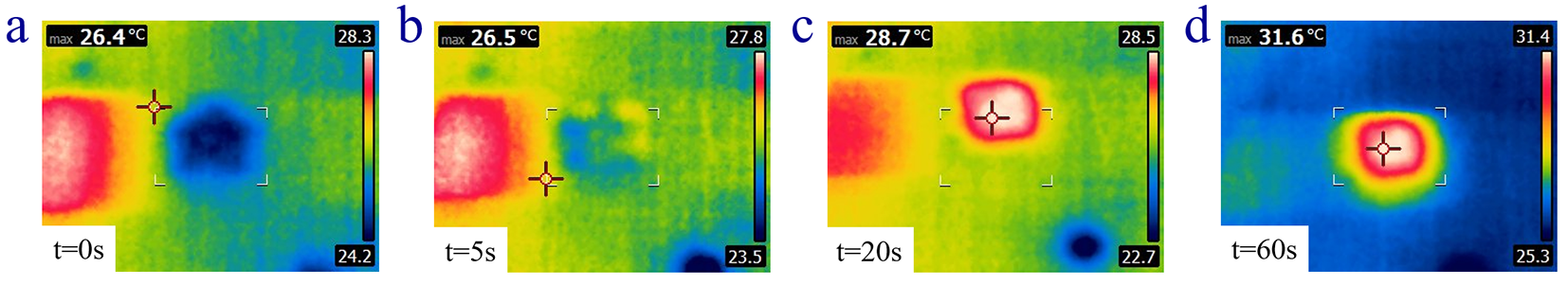

Supplement: Supplementary Materials — Figure S1: optical microscopic images of helical microfibers with different helical pitches. Scale bar: 450 μm. Figure S2: the Raman spectra of Ca-Alg microfibers and Ca-Alg microfibers coated with MXene. Figure S3: the FTIR spectra of Ca-Alg microfibers and Ca-Alg microfibers with MXene encapsulation. Figure S4: conductivity performances of microfibers. Figure S5: stress-strain curve of the pure straight microfiber. Figure S6: the progress of straight microfibers with MXene encapsulation under NIR irradiation. Figure S7: the progress of straight microfibers without MXene encapsulation under NIR irradiation. [file 7065907.f1.zip › Figure S7.png]
